# Supplementary material for: Patient-specific mutations impair BESTROPHIN1’s essential role in mediating Ca2+-dependent Cl- currents in human RPE
Source: eLife. 2017 Oct 24;6:e29914. doi: 10.7554/eLife.29914 (PMC5655127; doi:10.7554/eLife.29914)
Supplement: Figure 1—source data 1. — Ca2+-dependent Cl- current amplitudes in two clonal iPSC-RPEs (for WT and I201T) or iPSC-RPEs generated by two different sets of differentiations (for P274R) from the same donors. n = 5–6 for each data set. Diff: differentiation. [file elife-29914-fig1-data1.docx]

**Figure 1‒source data 1. Comparison of different data sets from the same donors.** Ca^2+^-dependent Cl^-^ current amplitudes in two clonal iPSC-RPEs (for WT and I201T) or iPSC-RPEs generated by two different sets of differentiations (for P274R) from the same donors. n= 5-6 for each data set. Diff: differentiation.

|  | 0 Ca^2+^ | | 1.2 μM Ca^2+^ | |
| --- | --- | --- | --- | --- |
|  | Clone #1 | Clone #2 | Clone #1 | Clone #2 |
| WT (pA/pF) | 4.2 ± 1.2 | 3.3 ± 0.9 | 358.9 ± 26.7 | 320.1 ± 64.3 |
| I201T (pA/pF) | 2.0 ± 0.3 | 3.2 ± 1.1 | 90.3 ± 48.3 | 102.8 ± 33.5 |
|  | | | | |
|  | Diff #1 | Diff #2 | Diff #1 | Diff #2 |
| P274R (pA/pF) | 2.1 ± 1.6 | 1.6 ± 1.5 | 5.5 ± 3.3 | 3.8 ± 1.0 |
