# Supplementary material for: Patient-specific mutations impair BESTROPHIN1’s essential role in mediating Ca2+-dependent Cl- currents in human RPE
Source: eLife. 2017 Oct 24;6:e29914. doi: 10.7554/eLife.29914 (PMC5655127; doi:10.7554/eLife.29914)
Supplement: Figure 8—source data 1. — aStatistics for the highest-resolution shell are shown in parentheses. [file elife-29914-fig8-data1.docx]

**Figure 8‒source data 1. Data collection and refinement statistics of L177T KpBest^ΔC11^**

| Wavelength (Å) | 0.97918 |
| --- | --- |
| Resolution range (Å) | 65.73 - 3.14 (3.24 - 3.14) ^a^ |
| Space group | *P* 2_1_2_1_2_1_ |
| Cell dimensions |  |
| a, b, c (Å) | 114.5, 160.6, 162.7 |
| α, β, γ (°) | 90, 90, 90 |
| Unique reflections | 53003 (5204) |
| Completeness (%) | 99.88 (99.50) |
| Mean I/sigma(I) | 11.91 (1.15) |
| Redundancy | 7.4 (7.6) |
| R_merge_/R_meas_/R_pim_ (CC_1/2_) | 0.061/0.086/0.061 (0.429) |
| Reflections used in refinement | 53002 (5202) |
| Reflections used for R-free | 1998 (197) |
| R-work / R-free | 0.199 / 0.237 |
| Number of non-hydrogen atoms | 10678 |
| Macromolecules (Protein) | 10622 |
| Zn | 15 |
| H_2_O | 41 |
| Protein residues | 1346 |
| RMS (bonds) | 0.002 |
| RMS (angles) | 0.51 |
| Ramachandran favored (%) | 97.9 |
| Ramachandran allowed (%) | 2.1 |
| Wilson B-factor | 88.64 |
| Average B-factor | 92.13 |
| Macromolecules (Protein) | 92.24 |
| Zn | 88.75 |
| H_2_O | 65.27 |
| PDB code | 5X87 |

^a^Statistics for the highest-resolution shell are shown in parentheses.
